# Combining Correlated Outcomes and Surrogate Endpoints in a Network Meta-Analysis of Colorectal Cancer Treatments

**DOI:** 10.3390/cancers12092663

**Published:** 2020-09-18

**Authors:** Tung Hoang, Jeongseon Kim

**Affiliations:** Department of Cancer Biomedical Science, National Cancer Center Graduate School of Cancer Science and Policy, Goyang 10408, Korea; 75256@ncc.re.kr

**Keywords:** network meta-analysis, colorectal cancer, surrogate endpoints

## Abstract

**Simple Summary:**

Currently, cytotoxic agents and biological targeted agents are commonly combined for the treatment of advanced or metastatic colorectal cancer. However, questions of ‘which chemotherapy or targeted therapy provides the higher efficacy and lower toxicity’ or ‘whether the addition of targeted therapy to chemotherapy not only increases the treatment effect but also reduces the adverse events’ have been raised. In this study, we firstly calculated the treatment effect on overall survival, which has not been reached in several randomized controlled trials, based on treatment effects on overall response rate and/or progression-free survival. Then we performed the network meta-analysis to compare the efficacy and safety of 12 commonly used regimens. Finally, our analyses showed that FOLFOX+cetuximab and FOLFIRI+bevacizumab have high probabilities of being first-line and second-line treatments in terms of efficacy and safety, respectively.

**Abstract:**

This study aimed to investigate the efficacy and safety of systemic therapies in the treatment of unresectable advanced or metastatic colorectal cancer. Predicted hazard ratios (HRs) and their 95% credible intervals (CrIs) for overall survival (OS) were calculated from the odds ratio (OR) for the overall response rate and/or HR for progression-free survival using multivariate random effects (MVRE) models. We performed a network meta-analysis (NMA) of 49 articles to compare the efficacy and safety of FOLFOX/FOLFIRI±bevacizumab (Bmab)/cetuximab (Cmab)/panitumumab (Pmab), and FOLFOXIRI/CAPEOX±Bmab. The NMA showed significant OS improvement with FOLFOX, FOLFOX+Cmab, and FOLFIRI+Cmab compared with that of FOLFIRI (HR = 0.84, 95% CrI = 0.73–0.98; HR = 0.76, 95% CrI = 0.62–0.94; HR = 0.80, 95% CrI = 0.66–0.96, respectively), as well as with FOLFOX+Cmab and FOLFIRI+Cmab compared with that of FOLFOXIRI (HR = 0.69, 95% CrI = 0.51–0.94 and HR = 0.73, 95% CrI = 0.54–0.97, respectively). The odds of adverse events grade ≥3 were significantly higher for FOLFOX+Cmab vs. FOLFIRI+Bmab (OR = 2.34, 95% CrI = 1.01–4.66). Higher odds of events were observed for FOLFIRI+Pmab in comparison with FOLFIRI (OR = 2.16, 95% CrI = 1.09–3.84) and FOLFIRI+Bmab (OR = 3.14, 95% CrI = 1.51–5.89). FOLFOX+Cmab and FOLFIRI+Bmab showed high probabilities of being first- and second-line treatments in terms of the efficacy and safety, respectively. The findings of the efficacy and safety comparisons may support the selection of appropriate treatments in clinical practice. PROSPERO registration: CRD42020153640.

## 1. Background

Over the past few decades, colorectal cancer (CRC) has been a global public health issue, with an estimated 1.8 million cases newly diagnosed in 2018 [1]. It is still the second most common cancer in men and the third most common cancer in women. Approximately 25% of CRC patients have metastatic disease at the time of diagnosis, and metastases develop in approximately 20–50% of people who have undergone surgical treatment for the early stage of CRC [2].

In clinical practice, systemic treatments, which commonly combine cytotoxic agents and biological targeted agents to optimize the treatment effects, are proposed for unresectable metastatic CRC (mCRC) [2]. The National Comprehensive Cancer Network (NCCN) guidelines recommend chemotherapy options for people with mCRC, including 5-fluorouracil and folinic acid, in combination with oxaliplatin (FOLFOX), irinotecan (FOLFIRI), oxaliplatin and irinotecan (FOLFOXIRI), and capecitabine in combination with oxaliplatin (CAPEOX) [3,4]. These regimens are introduced and then combined with bevacizumab (Bmab) [5], a monoclonal antibody against vascular endothelial growth factor (VEGF), to increase their activity [3,4]. In patients with mCRC harboring mutations in exons 2, 3, and 4 of the Kirsten rat sarcoma (*KRAS*) and neuroblastoma rat sarcoma (*NRAS*) genes, which belong to oncogenes from the rat sarcoma (*RAS*) gene family, cetuximab (Cmab) and panitumumab (Pmab) are suggested to be combined with the FOLFOX or FOLFIRI regimen [3,4,6,7]. These two anti-epidermal growth factor receptor (EGFR) treatments require confirmation of *RAS* wild-type before administration. 

In clinical practice, questions regarding which chemotherapy or targeted therapy provides higher efficacy and lower toxicity, and whether the addition of targeted therapy to chemotherapy not only increases the treatment effect but also reduces adverse events (AEs), have been raised. A previous network meta-analysis (NMA) of 38 various regimens provided the treatment ranking results to suggest first-line or second-line treatment [8]. However, the data on overall survival (OS), which is known to be the gold standard in clinical trials [9], have not been available for all treatments. Given that findings of various outcomes in the previous NMA may make it difficult to directly answer questions regarding the efficacy and safety, we shifted our interest to calculating the predicted hazard ratio (HR) and 95% credible interval (CrI) for OS based on other surrogate endpoints, including the overall response rate (ORR) and/or progression-free survival (PFS), combined with the observed HR for OS as the efficacy and the odds ratio (OR) for AEs grade ≥3 as safety. Additionally, we performed an NMA of randomized controlled trials (RCTs) to suggest candidate treatments.

## 2. Methods

### 2.1. Data Sources

We used data from the previous systematic review and NMA, which included treatment effect sizes from 94 previous RCTs for advanced/metastatic (a/m)CRC [8]. In the current study, RCTs that did not include the following treatments were excluded: FOLFOX/FOLFIRI±Bmab/Cmab/Pmab and CAPEOX/FOLFOXIRI±Bmab [8]. Studies that did not report all ORR, PFS, OS, or AEs grade ≥3 outcomes were also excluded. ORR was defined based on the Response Evaluation Criteria in Solid Tumors guidelines [10] or the World Health Organization recommendations [11]. PFS was defined as the time frame from randomization to objective progression, death, or last tumor assessment without progression before any additional anticancer therapy, whichever occurred first [12]. OS was defined as the time frame from randomization to death or the last date that the patient was known to be alive for those not known to have died, whichever occurred first [12]. As a result, a total of 49 studies from 45 RCTs were included in the final analysis [13,14,15,16,17,18,19,20,21,22,23,24,25,26,27,28,29,30,31,32,33,34,35,36,37,38,39,40,41,42,43,44,45,46,47,48,49,50,51,52,53,54,55,56,57,58,59,60,61].

### 2.2. Statistical Analyses

#### 2.2.1. Multivariate Random Effects Model for Surrogate Endpoints

We applied the trivariate random effects model, which was developed and validated by Bujkiewiecz et al., to estimate the joint treatment effects of final outcomes on surrogate endpoints [62]. In general, treatment effects on OS and PFS were presented by calculating HRs, and the treatment effect on ORR was obtained by computing ORs. In the current study, $Y_{1i}$ representing log OR on ORR, $Y_{2i}$ representing log HR on PFS, and $Y_{3i}$ representing log HR on OS are assumed to be correlated and normally distributed [63]:

$$\left(\begin{array}{c} Y_{1i}\\ Y_{2i}\\ Y_{3i} \end{array}\right)∼N(\left(\begin{array}{c} \mu_{1i}\\ \mu_{2i}\\ \mu_{3i} \end{array}\right),\Sigma i),\Sigma i=\left(\begin{array}{ccc} \sigma_{1i}^{2} & \sigma_{1i}\sigma_{2i}\rho_{wi}^{12} & \sigma_{1i}\sigma_{3i}\rho_{wi}^{13}\\ \sigma_{2i}\sigma_{1i}\rho_{wi}^{12} & \sigma_{2i}^{2} & \sigma_{2i}\sigma_{3i}\rho_{wi}^{23}\\ \sigma_{3i}\sigma_{1i}\rho_{wi}^{13} & \sigma_{3i}\sigma_{2i}\rho_{wi}^{23} & \sigma_{3i}^{2} \end{array}\right)$$

where $\begin{array}{c} \mu_{ki} \end{array}$ are the true treatment effects, $\sigma_{ki}^{2}$ are the corresponding variances of treatment effects for individual study i and outcome k, and $\rho_{wi}^{kl}$ are within-study correlations among these estimates. The between-study variability is estimated by modeling $\begin{array}{c} \mu_{ki} \end{array}$ in a conditional univariate normal distribution with structured covariance [63]:

$$\;\mu_{1i}\;\sim \;N\;(\eta_{1},\;\psi_{1}^{2})$$

$$\;\mu_{2i}\;|\;\mu_{1i}\;\sim \;N\;(\eta_{2i},\;\psi_{2}^{2})$$

$$\;\eta_{2i}=\;\lambda_{20}+\;\lambda_{21}\;\mu_{1i}$$

$$\;\mu_{3i}\;|\;\mu_{2i}\;\sim \;N\;(\eta_{3i},\;\psi_{3}^{2})$$

$$\;\eta_{3i}=\;\lambda_{30}+\;\lambda_{32}\;\mu_{2i}$$

where the variances $\psi_{k}^{2}$ are related to the between-study heterogeneity parameters $\tau_{k}^{2}$ through the regression coefficients $\;\lambda_{kl}$, which are related to both $\tau_{k}^{2}$ and between-study correlations $\rho_{b}^{kl}$. Given that HR of OS is positively associated with HR of PFS and negatively associated with OR of ORR, we allocated uniform prior distributions for the between-study correlations, $\rho_{b}^{13}\;\sim \;U\left(-1,0\right)$ and $\rho_{b}^{23}\;\sim \;U\left(0,1\right)$. Additionally, we assigned half-normal distributions for heterogeneity parameters, $\tau_{k}\;\sim \;N\left(0,1000\right)I\left(0,\right)$, and normal distributions for other parameters, $\eta_{1},\;\lambda_{20},\;\lambda_{30}\;\sim \;N\left(0,1000\right)$.

For studies reporting OS and a single treatment effect of ORR or PFS only, the reduced model of bivariate random effects [63] was similarly applied to investigate the association between OS and ORR or between OS and PFS.

The estimated parameters were borrowed from multivariate random effects (MVRE) models, and the predicted log HR for OS was calculated for studies reporting OR for ORR and/or HR for PFS but not HR for OS.

#### 2.2.2. Network Meta-Regression Analysis of Treatment Therapies

In the network meta-analysis, we calculated the pooled HR of OS and the pooled OR of AEs grade ≥3 to compare the pairwise efficacy and safety between mCRC treatments, following a generalized linear model [64,65]:

$$\theta_{ik}=\mu_{i}+\delta_{ik}+(\beta_{1,t_{ik}}-\beta_{1,t_{i1}})x_{i}$$

where the trial-specific effects of treatment in arm 1 of trial *i* denote $\mu_{i}$, the trial-specific effects of treatment in arm *k* compared with the treatment in arm 1 in the same trial denote $\;\delta_{ik}$, and $t_{ik}$ and $t_{1k}$ are the treatments in arm k and arm 1 of trial i. The trial-level subgroup indicator $x_{i}$ is defined as

$$x_{i}=\{\begin{array}{c} 1\;if\;study\;i\;compares\;primary\;treatments\\ 0\;if\;sudy\;i\;compares\;secondary\;treatments \end{array}$$

Furthermore, the consistency assumption between direct and indirect estimates and the between-study heterogeneity were evaluated by conducting the node-splitting statistic and calculating the *I*^2^ values [66].

We additionally ranked the treatment based on the surface under the cumulative ranking curve (SUCRA) values [67]. The SUCRA value for treatment i is defined as follows:

$$SUCRA\left(i\right)=\frac{\sum_{k=1}^{n-1}F\left(i,k\right)}{n-1}$$

where n is the number of treatments, and F(i,k) is the cumulative probability that treatment i ranks kth best and is calculated as

$$F\left(i,k\right)=\overset{k}{\underset{j=1}{\sum }}P\left(i,j\right)$$

where *P*(*i*,*j*) is the probability that treatment *i* ranks jth for a particular outcome of OS and AEs grade ≥3.

The SUCRA value is therefore a representative number of the overall ranking, which ranges from 0 to 1 [67]. A higher SUCRA value indicates a higher probability of the efficacy or safety endpoint [67]. The SUCRA values were standardized and presented in a two-dimensional plot according to the efficacy and safety outcomes. We then applied the k-means clustering method to group treatments showing high efficacy and safety, high efficacy and low safety, high safety and low efficacy, and low efficacy and safety [68].

All the models regarding the Bayesian approach were performed in WinBUGS 1.4.3 (MRC Biostatistics Unit, UK) [69], using 3 chains and 150,000 iterations of the Markov chain Monte Carlo simulation process (including 50,000 burn-in iterations).

The study methodology and progress were registered and approved by the National Institute for Health Research—an international prospective register of systematic reviews (PROSPERO registration number: CRD42020153640).

## 3. Results

### 3.1. Association between Surrogacy Endpoint and Correlated Outcome

The characteristics and findings from 49 included studies are summarized in Table 1. Bivariate and trivariate random effect models were carried out for the surrogacy associations between treatment effect sizes. Then, we calculated the predicted HRs (95% CIs) for the OS of 17 and five study populations that reported results for ORR only and both ORR and PFS, respectively (Table 1). The predicted OS was not significantly different for all 22 pairwise treatment comparisons.

Table 2 shows the surrogacy parameters of the MVRE models. The 95% CrIs of posterior intercepts containing zero confirmed that no treatment effect on a surrogate endpoint(s) suggested no treatment effect on the outcome. In other words, in studies with no significant differences between the intervention and comparison groups in terms of ORR and/or PFS, there were no differences in OS between the groups either. 

When the 95% CrI posterior slope did not contain a zero, positive slopes indicated significant positive associations and negative slopes indicated significant negative associations between treatment effects on surrogate endpoints and the outcome. As a result, the treatment effect on OS was observed to be significantly positively associated with the treatment effect on PFS (posterior slope = 0.79, 95% CrI = 0.49–1.09), and the adjusted R-squared value was relatively high (posterior R-squared = 0.54, 95% CrI = 0.25–0.76), with somewhat low variance (posterior variance = 0.02, 95% CrI = 0.01–0.04) in the bivariate model. Although there was a significant negative association between the treatment effects on OS and ORR, the relationship was not strong, with the upper limit of the slope (−0.04) and the lower limit of the R-squared value (0.01) close to zero and somewhat high variance (posterior variance = 0.07, 95% CrI = 0.04–0.13). In the trivariate model, we observed a strong relationship between the treatment effects on OS and both ORR and PFS (posterior slope = 0.76, 95% CrI = 0.46–1.06; posterior R-squared = 0.53, 95% CrI = 0.22–0.76; posterior variance = 0.04, 95% CrI = 0.02–0.07), which was similar to that of the bivariate model of PFS as the surrogate endpoint.

### 3.2. Network Geometry for the Efficacy and Safety of CRC Treatments

**Figure 1:**
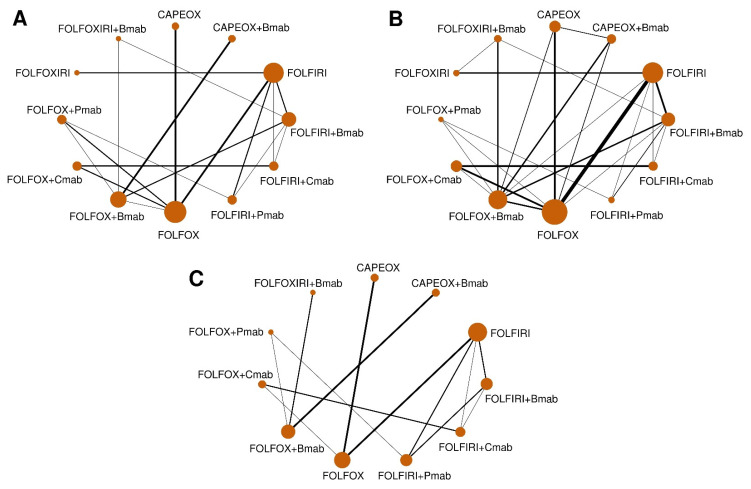
Network geometry of head-to-head trials. Data are presented as networks for comparison of (**A**) overall survival (observed values only), (**B**) overall survival (combining observed and predicted values), and (**C**) adverse events grade ≥3. The width of each line reflects the number of studies. The sizes of the circles are proportional to the number of study participants. FOLFOX (5-fluorouracil, folinic acid, and oxaliplatin), FOLFIRI (5-fluorouracil, folinic acid, and irinotecan), FOLFOXIRI (5-fluorouracil, folinic acid, oxaliplatin, and irinotecan), CAPEOX (capecitabine and oxaliplatin), Bmab (bevacizumab), Cmab (cetuximab), and Pmab (panitumumab).

A total of 12 commonly used regimens for a/mCRC were included in the NMA for efficacy (Figure 1A). After including the predicted estimates from MVRE models, there was new evidence of the direct comparison between FOLFOX+Bmab and FOLFOX+Cmab, FOLFOXIRI+Bmab and FOLFOXIRI, and CAPEOX and CAPEOX+Bmab (Figure 1B). The NMA for safety included 11 regimens, except for FOLFOXIRI, because data for comparative AEs grade ≥3 for FOLFOXIRI were not available from RCTs (Figure 1C).

### 3.3. Pairwise Treatment Effect of Included Regimens

The comparative treatment effects on OS as efficacy and AEs grade ≥3 as safety are provided in Table 3. On the one hand, NMAs showed significant OS improvements with FOLFOX, FOLFOX+Cmab, and FOLFIRI+Cmab in comparison with FOLFIRI, with HR = 0.84, 95% CrI = 0.73–0.98; HR = 0.76, 95% CrI = 0.62–0.94; HR = 0.80, 95% CrI = 0.66–0.96, respectively. When irinotecan or oxaliplatin in the FOLFOXIRI regimen was replaced with Cmab, the results showed an improvement in OS by 31% (HR = 0.69, 95% CrI = 0.51–0.94) or 27% (HR = 0.73, 95% CrI = 0.54–0.97), respectively. On the other hand, the rates of AEs grade ≥3 were significantly higher among patients treated with FOLFOX+Cmab than among those treated with FOLFIRI+Bmab (OR = 2.34, 95% CrI = 1.01–4.66). Additionally, higher odds of events were observed when adding Pmab into the FOLFIRI regimen (OR = 2.16, 95% CrI = 1.09–3.84 for FOLFIRI+Pmab vs. FOLFIRI) or replacing Bmab with Pmab in the FOLFIRI+Bmab regimen (OR = 3.14, 95% CrI = 1.51–5.89 for FOLFIRI+Pmab vs. FOLFIRI+Bmab).

The test for consistency assumption showed that there was no significant difference between direct and indirect evidence (*p* > 0.05, Table 4). Additionally, substantial heterogeneity was observed for safety outcome (pairwise *I*^2^ = 71% and consistent *I*^2^ = 76%) but not for efficacy outcome (pairwise *I*^2^ = 29% and consistent *I*^2^ = 36%).

### 3.4. Investigation of Treatment Ranking

**Figure 2:**
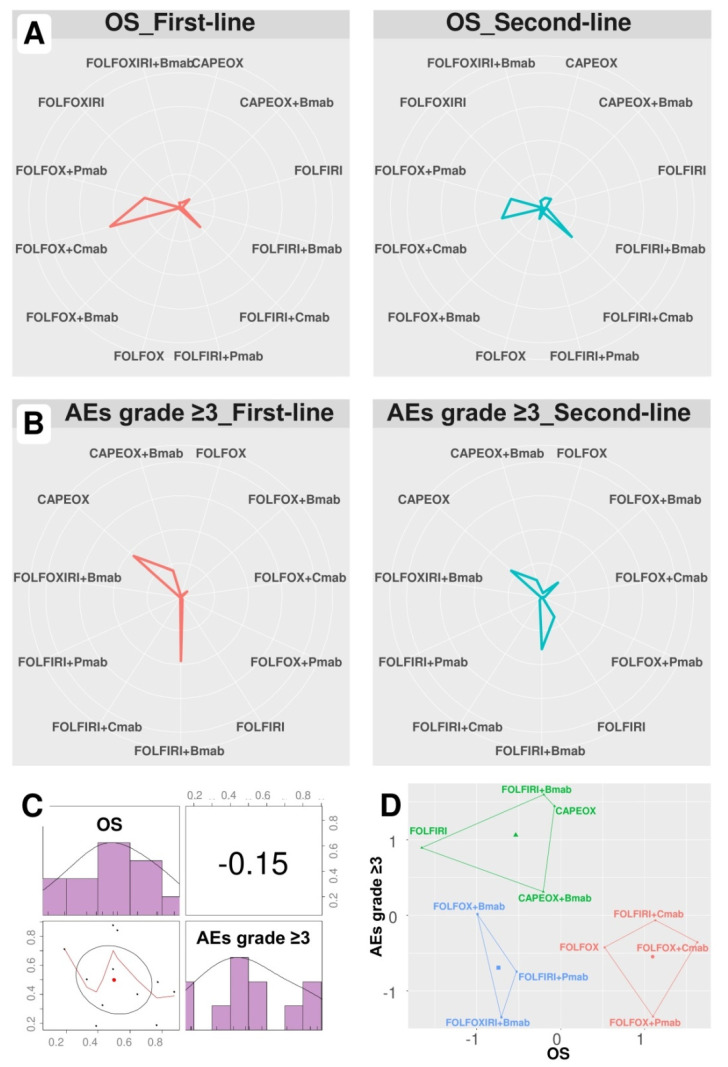
Treatment ranking probability and SUCRA ranking plots. Data are presented as the probability of first- and second-line treatment ranking according to (**A**) overall survival and (**B**) adverse events grade ≥ 3. (**C**) Correlation and (**D**) k-means clustering analysis of SUCRA values. FOLFOX (5-fluorouracil, folinic acid, and oxaliplatin), FOLFIRI (5-fluorouracil, folinic acid, and irinotecan), FOLFOXIRI (5-fluorouracil, folinic acid, oxaliplatin, and irinotecan), CAPEOX (capecitabine and oxaliplatin), Bmab (bevacizumab), Cmab (cetuximab), Pmab (panitumumab), OS (overall survival), and AEs (adverse events).

Figure 2A,B show the probability of regimens becoming first- and second-line treatments, and other-line treatment probabilities are detailed in Table 5 and Table 6. As a result, while FOLFOX+Cmab had the highest probabilities of being primary (43.3%) and secondary (24.6%) treatments in terms of OS, FOLFIRI+Bmab had the highest probabilities of being both primary and secondary treatments in terms of OS (37.8% and 30.8%, respectively) for AEs grade ≥ 3. Additionally, FOLFOX+Cmab and CAPEOX also had high probabilities of being a secondary treatment regarding OS (24.6%) and primary treatment regarding AEs grade ≥ 3 (37.1%), respectively. 

As shown in Table 7, FOLFOX+Cmab was the most effective treatment regimen (SUCRA = 0.88), whereas FOLFIRI+Bmab was the safest treatment regimen (SUCRA = 0.87). The SUCRA values of regimens for cumulative ranking probabilities between OS and AEs grade ≥ 3 were slightly correlated (correlation coefficient −0.15, Figure 2C).

K-means clustering analysis identified three clusters based on SUCRA values (Figure 2D): low efficacy and high safety (FOLFIRI±Bmab and CAPEOX±Bmab), low efficacy and safety (FOLFOX/FOLFOXIRI+Bmab and FOLFIRI+Pmab), and high efficacy and low safety (FOLFOX±Pmab and FOLFOX/FOLFIRI Cmab).

## 4. Discussion

This study applied MVRE models to calculate the predicted treatment effect on the correlated outcome of OS based on treatment effects on surrogated endpoints, including ORR and/or PFS. Both the observed and predicted HRs for OS as the efficacy and ORs for AEs grade ≥ 3 as safety were included in the Bayesian framework of NMA, which is based on both direct and indirect comparisons. Our findings indicated the high probabilities of FOLFOX+Cmab becoming a primary and secondary treatment in terms of efficacy and FOLFIRI+Bmab becoming primary and secondary treatment in terms of safety in the treatment of a/mCRC. 

Although OS is the meaningful gold standard in oncology research and practice, surrogate endpoints have also been investigated over the past few decades because of the limitation of obtaining OS. It was reported that 84% of trials used surrogate endpoints during 2005–2012 [70] and 66% oncology indications between 2009 and 2014 [71] were approved by the United States Food and Drug Administration. In mCRC, PFS was mostly evaluated for the prognosis of OS outcome, in addition to response rate and tumor shrinkage criteria [72,73,74,75,76]. Cicero et al. recently found a moderate correlation between OS and PFS in first-line and second-line treatments with FOLFOX+Bmab for mCRC [72]. Similar findings were presented in a systematic review of twenty individual RCTs in the second-line treatment of mCRC, with moderate (0.73) and poor (0.17) correlations of PFS and ORR with OS, respectively [73]. However, the surrogacy relationships, which were performed by Bujkiewicz et al. in the Bayesian framework [62], were determined to have the advantage of considering the uncertainty of measurement errors of treatment effect on surrogate endpoints and allowed us to combine both treatments on ORR and PFS in the calculation of HR for OS [63]. In the present study, we also observed that the association between HR for OS and HR for PFS (R-squared = 0.54, 95% CrI = 0.25–0.76) was not much improved when adding OR for ORR as the second surrogate endpoint by applying the MVRE models (R-squared = 0.53, 95% CrI = 0.22–0.76).

In contrast, several studies have questioned the accuracy of surrogate endpoints in the prediction of treatment outcomes especially in oncology research [77]. A lack of validation due to weak to moderate correlations between ORR or PFS and OS was reported in patients with cancer treated with immune checkpoint inhibitors [77]. Additionally, a cross-sectional study of 51 products (26 products were assessed through conditional marketing authorization, and 25 products were assessed through accelerated assessment), regardless of treatment indications, which were authorized between 2011 and 2018 by the European Medicines Agency found that 46 approvals were based on surrogate endpoints which had not been demonstrated to obviously predict clinical outcomes [78]. In patients with mCRC, although PFS has been examined as a surrogate endpoint of OS in either literature-based (50 RCTs) [79] or individual patient-based (22 RCTs) [80] analysis, further studies of individual patient data at different time points are needed to validate the findings.

The combinations of 5-fluorouracil and folinic acid with oxaliplatin or irinotecan which were first launched in the 1990s showed a significant improvements in the response rate and survival time compared with those of regimens without oxaliplatin or irinotecan [81]. Several RCTs have been conducted to directly compare the activity of FOLFOX and FOLFIRI since then [51,53,58]. While FOLFOX was reported to be associated with an approximately 30% risk of death [51,58], another head-to-head trial [53], as well as our predicted values from RCTs reporting surrogate endpoints [56,59], showed comparative effects. The current NMA supported the superior efficacy of FOLFOX over FOLFIRI, while the safety was still equivalent. However, the choice of oxaliplatin-based or irinotecan-based therapy remains controversial. Physicians might prefer FOLFOX due to the consideration of the significant cost-effectiveness and the lower nausea and vomiting side effects than FOLFIRI, which might not be appropriate for older female patients [82]. In contrast, hand-foot syndrome is more frequent in patients treated with FOLFOX, which might not be preferred in some polar countries. Among European countries, the preference of using FOLFOX- and FOLFIRI-containing regimens in first-line and second-line treatments was also reversed between Germany–Spain and Italy–France [83]. Nevertheless, our NMA showed that the efficacy and safety between FOLFOX- and FOLFIRI-containing regimens (FOLFOX+Bmab vs. FOLFIRI+Bmab, FOLFOX+Cmab vs. FOLFIRI+Cmab, and FOLFOX+Pmab vs. FOLFIRI+Pmab) were not significantly different.

While including anti-VGFR therapy such as Bmab in chemotherapy regimens was introduced to exhibit significant benefits on OS in the ARTIST trial [38], the treatment effect on OS was not reported in other trials [17,25,34,57]. A previous NMA reported consistent findings of comparable OS and AEs grade ≥3 for FOLFOX/FOLFIRI/FOLFOXIRI/CAPEOX plus Bmab vs. chemotherapy alone, although FOLFOX/FOLFIRI plus Bmab resulted in a significantly better disease control rate and PFS than did FOLFOX/FOLFIRI [8]. Wu et al. recently reported a nonsignificant difference in OS between chemotherapy+Bmab and chemotherapy alone in the subset of KRAS (HR = 1.17, 95% CI = 0.93–1.48) and RAS wild-type (HR = 0.88, 95% CI = 0.63–1.23) mCRC patients, despite the small number of individual studies [84].

In this study, we did not observe any significant differences in OS among subjects who received chemotherapy plus Bmab, Cmab, or Pmab. However, pooled analysis for the CRC side suggested Cmab and Pmab for left-sided mCRC treatments and Bmab for right-sided mCRC treatments [84]. Additionally, the effect of anti-EGFR therapies was different according to the presence of KRAS or NRAS mutations [85]. While Cmab and Pmab showed a significant prolongation of OS or PFS among RAS wild-type mCRC patients, the survival outcomes tended to be worse among patients with RAS mutations [85]. The addition of VEGF or EGFR inhibitors into chemotherapy showed similar effects in the first-line treatment of nonmutated RAS mCRC [85]. Cmab- and Pmab-based therapies revealed significant improvements in OS of 25% and 32%, respectively, compared with chemotherapy+Bmab among subjects harboring KRAS wild-type but not RAS wild-type subjects [84].

In the present study, we took the strengths of MVRE models that take into account surrogate endpoints in the final clinical outcome. Despite the consistency of the treatment effects with the previous NMA [8], the treatment effects tended to be close to the null hypothesis in our study because we additionally considered the treatment effect on OS from RCTs that did not report the HR on OS or the HR was not reached. We additionally considered whether the treatment was used for the primary or secondary indication in the meta-regression model to justify the effect of the treatment line.

Despite its strengths, the study has some limitations. Subgroup analyses of cancer side-specific or genotype were not evaluated in the current study. We also combined the treatments based on the components of the regimens, regardless of the schedule (sequentially or continuously, doses, and orders) and drug administration routes (bolus or infusion). Although the dose reduction due to side effects was reported to not have an effect on survival for chemotherapy treatments of colon cancer, we were unable to investigate the impact of these parameters [86]. Furthermore, the types or choices of chemotherapy can depend on the site or a regional preference of the hospital when standard cares show no differences. Finally, publication bias was not assessed because of the small number of head-to-head RCTs for each treatment comparison.

## 5. Conclusions

In summary, we found a significant relationship between the correlated outcome of the treatment effect on OS and surrogated endpoints. The findings of efficacy and safety comparisons may support the selection of appropriate treatments in clinical practice. 

## Figures and Tables

**Table 1:** Individual study characteristics and treatment effect predictions on correlated outcome.

Study	Treatment Line	Treatment Arms		OR (95% CI) for Overall Response Rate	HR (95% CI) for Progression-Free Survival	Observed HR (95% CI) for Overall Survival	Predicted HR (95% CrI) for Overall Survival	OR (95% CI) for Adverse Events Grade ≥3
		Intervention Arm	Comparison Arm					
Uetake 2018 et al. [13]	1-line	FOLFOX+Cmab	FOLFOX+Bmab	**2.94 (1.16–7.47)**	0.80 (0.51–1.26)		0.81 (0.54–1.21)	
Qin 2018 et al. [14]	1-line	FOLFOX+Cmab	FOLFOX	**2.89 (1.92–4.36)**	**0.63 (0.50–0.79)**	**0.76 (0.61–0.95)**		
Maiello 2018 et al. [15]	1-line	FOLFOX+Bmab	CAPEOX+Bmab	1.34 (0.65–2.76)	0.96 (0.65–1.41)	1.21 (0.77–1.92)		
Hurwitz 2018 et al. [16]	1-line	FOLFOXIRI+Bmab	FOLFOX+Bmab	1.60 (0.95–2.71)	**0.68 (0.50–0.92)**		0.72 (0.48–1.07)	
Hou 2018 et al. [17]	1-line	FOLFOXIRI+Bmab	FOLFOXIRI	**2.41 (1.12–5.18)**			0.82 (0.47–1.42)	
Gomez 2018 et al. [18]	1-line	FOLFOX+Bmab	FOLFOXIRI+Bmab					**0.51 (0.32–0.81)**
Schmoll 2017 et al. [19]	1-line	FOLFOX+Bmab	FOLFOXIRI+Bmab	0.64 (0.37–1.08)	0.80 (0.61–1.04)		0.81 (0.54–1.21)	
Carrato 2017 et al. [20]	1-line	FOLFOX+Pmab	FOLFIRI+Pmab	1.40 (0.52–3.74)	0.90 (0.60–1.50)	1.00 (0.60–1.80)		1.60 (0.51–5.04)
Yamazaki 2016 et al. [21]	1-line	FOLFIRI+Bmab	FOLFOX+Bmab	1.07 (0.70–1.64)	0.91 (0.72–1.13)	0.99 (0.79–1.25)		
Shitara 2016 et al. [22]	2-line	FOLFIRI+Pmab	FOLFIRI+Bmab	**14.3 (3.95–51.7)**				
Ciardiello 2016 et al. [23]	2-line	FOLFOX+Cmab	FOLFOX	1.90 (0.80–4.52)	0.81 (0.58–1.12)	0.57 (0.32–1.02)		
Tournigand 2015 et al. [24]	1-line	FOLFOX+Bmab	CAPEOX+Bmab		0.98 (0.74–1.31)	1.24 (0.98–1.59)		0.63 (0.39–1.02)
Passardi 2015 et al. [25]	1-line	FOLFOX+Bmab	FOLFOX	1.29 (0.76–2.19)			0.85 (0.49–1.47)	
		FOLFIRI+Bmab	FOLFIRI	0.74 (0.39–1.41)			0.96 (0.55–1.68)	
Hecht 2015 et al. [26]	2-line	FOLFIRI+Pmab	FOLFIRI+Bmab	1.99 (0.98–4.03)	1.01 (0.68–1.50)	1.06 (0.75–1.49)		**2.20 (1.06–4.56)**
Gruenberger 2015 et al. [27]	1-line	FOLFOXIRI+Bmab	FOLFOX+Bmab	2.58 (0.94–7.05)	**0.43 (0.26–0.72)**	**0.35 (0.15–0.80)**		3.68 (0.69–19.5)
Cremolini 2015 et al. [28]	1-line	FOLFIRI+Bmab	FOLFOXIRI+Bmab	**0.63 (0.44–0.90)**	**0.77 (0.65–0.93)**	**0.80 (0.65–0.98)**		
Schwartzberg 2014 et al. [29]	1-line	FOLFOX+Pmab	FOLFOX+Bmab	1.20 (0.75–1.92)	0.87 (0.65–1.17)	**0.62 (0.44–0.89)**		2.02 (0.98–4.16)
Peeters 2014 et al. [30]	2-line	FOLFIRI+Pmab	FOLFIRI	**5.19 (3.29–8.19)**	**0.82 (0.69–0.97)**	0.92 (0.78–1.10)		
				0.89 (0.53–1.50)	0.94 (0.78–1.14)	0.93 (0.77–1.13)		
Peeters 2010 et al. [42]		FOLFIRI+Pmab	FOLFIRI					**2.46 (1.75–3.47)**
								**1.76 (1.22–2.53)**
Heinemann 2014 et al. [31]	1-line	FOLFIRI+Cmab	FOLFIRI+Bmab	1.18 (0.85–1.64)	1.06 (0.88–1.26)	**0.77 (0.62–0.96)**		1.40 (0.99–1.97)
Folprecht 2014 et al. [32]	1-line	FOLFOX+Cmab	FOLFIRI+Cmab		1.03 (0.66–1.61)	1.18 (0.79–1.74)		
Folprecht 2010 et al. [44]	1-line			1.62 (0.74–3.59)			0.81 (0.47–1.40)	0.81 (0.35–1.88)
Douillard 2014 et al. [33]	1-line	FOLFOX+Pmab	FOLFOX	**1.47 (1.08–2.01)**	**0.80 (0.67–0.95)**	0.88 (0.73–1.06)		
				0.97 (0.66–1.43)	**1.27 (1.04–1.55)**	1.17 (0.95–1.45)		
Cao 2014 et al. [34]	2-line	FOLFIRI+Bmab	FOLFIRI	**2.28 (1.14–4.56)**			0.75 (0.43–1.31)	0.56 (0.27–1.15)
Personeni 2013 et al. [35]	1-line	FOLFIRI	FOLFIRI+Cmab	0.98 (0.42–2.30)			0.90 (0.52–1.56)	
Van 2011 et al. [36]	1-line	FOLFIRI+Cmab	FOLFIRI	**1.40 (1.11–1.76)**	0.85 (0.73–1.00)	0.88 (0.77–1.00)		**2.46 (1.90–3.18)**
Masi 2011 et al. [37]	1-line	FOLFIRI	FOLFOXIRI		**0.59 (0.45–0.76)**	**0.74 (0.56–0.96)**		
Falcone 2007 et al. [50]	1-line	FOLFIRI	FOLFOXIRI	**0.43 (0.26–0.72)**			1.08 (0.60–1.95)	
Guan 2011 et al. [38]	1-line	FOLFIRI+Bmab	FOLFIRI	**2.62 (1.26–5.48)**	**0.44 (0.31–0.63)**	**0.62 (0.41–0.95)**		
Ducreux 2011 et al. [39]	1-line	CAPEOX	FOLFOX	0.75 (0.46–1.24)	1.00 (0.79–1.27)	1.02 (0.80–1.29)		
Cassidy 2011 et al. [40]	1-line	FOLFOX	CAPEOX			0.95 (0.84–1.07)		**1.42 (1.11–1.83)**
		FOLFOX+Bmab	CAPEOX+Bmab			0.95 (0.80–1.13)		**1.78 (1.22–2.61)**
Cassidy 2008 et al. [48]		FOLFOX	CAPEOX		0.96 (0.81–1.13)			
		FOLFOX+Bmab	CAPEOX+Bmab		1.01 (0.85–1.12)			
Vamvakas 2010 et al. [41]	1-line	FOLFIRI	FOLFOXIRI	0.67 (0.41–1.08)	1.15 (0.86–1.48)	1.08 (0.80–1.45)		
Ocvirk 2010 et al. [43]	1-line	FOLFOX+Cmab	FOLFIRI+Cmab	0.93 (0.49–1.77)	1.06 (0.74–1.52)	0.98 (0.67–1.44)		1.66 (0.87–3.16)
Bokemeyer 2009 et al. [45]	1-line	FOLFOX	FOLFOX+Cmab	0.66 (0.43–1.03)	0.93 (0.71–1.23)		0.91 (0.61–1.36)	0.73 (0.45–1.18)
Rothenberg 2008 et al. [46]	2-line	FOLFOX	CAPEOX	0.88 (0.59–1.31)	0.97 (0.83–1.14)	1.03 (0.87–1.23)		**3.25 (2.20–4.80)**
Hochster 2008 et al. [47]	1-line	FOLFOX	CAPEOX	**2.69 (1.11–6.55)**			0.73 (0.41–1.27)	1.15 (0.46–2.85)
		FOLFOX+Bmab	CAPEOX+Bmab	1.29 (0.67–2.48)			0.85 (0.49–1.47)	1.31 (0.67–2.58)
Porschen 2007 et al. [49]	1-line	CAPEOX	FOLFOX	0.79 (0.55–1.13)	1.17 (0.96–1.43)		1.08 (0.72–1.63)	
Goldberg 2006 et al. [51]	1-line	FOLFOX	FOLFIRI	**1.98 (1.25–3.16)**	**0.55 (0.43–0.70)**	**0.76 (0.60–0.97)**		
Polikoff 2005 et al. [52]	2-line	FOLFOX+Cmab	FOLFOX	**0.13 (0.05–0.34)**			1.40 (0.69–2.90)	
Kalofonos 2005 et al. [53]	1-line	FOLFIRI	FOLFOX	1.08 (0.66–1.76)			0.88 (0.51–1.53)	
Comella 2005 et al. [54]	1-line	FOLFIRI	FOLFOX	**0.58 (0.35–0.95)**			1.01 (0.57–1.80)	1.46 (0.91–2.35)
Colucci 2005 et al. [55]	1-line	FOLFIRI	FOLFOX	0.89 (0.57–1.38)		1.04 (0.80–1.37)		
Tournigand 2004 et al. [56]	1-line	FOLFIRI	FOLFOX	1.12 (0.66–1.90)			0.88 (0.51–1.52)	**0.40 (0.23–0.70)**
	2-line	FOLFOX	FOLFIRI	**3.83 (1.03–14.2)**			0.67 (0.37–1.20)	1.21 (0.63–2.30)
Hurwitz 2004 et al. [57]	1-line	FOLFIRI+Bmab	FOLFIRI	**1.52 (1.15–2.02)**			0.82 (0.47–1.42)	**0.50 (0.35–0.72)**
Goldberg 2004 et al. [58]	1-line	FOLFOX	FOLFIRI	**1.81 (1.27–2.58)**	**0.74 (0.61–0.89)**	**0.66 (0.54–0.82)**		
Rougier 2002 et al. [59]	2-line	FOLFIRI	FOLFOX	0.48 (0.13–1.82)			1.05 (0.59–1.89)	
NCT01374425 [60]	1-line	FOLFOX+Bmab	FOLFIRI+Bmab	0.83 (0.55–1.27)	0.79 (0.61–1.01)	0.76 (0.56–1.04)		
NCT00778830 [61]	1-line	FOLFIRI+Cmab	FOLFOX+Cmab	0.76 (0.47–1.24)			0.95 (0.54–1.67)	

OR (odds ratio), HR (hazard ratio), CI (confidence interval), CrI (credible interval), FOLFOX (5-fluorouracil, folinic acid, and oxaliplatin), FOLFIRI (5-fluorouracil, folinic acid, and irinotecan), FOLFOXIRI (5-fluorouracil, folinic acid, oxaliplatin, and irinotecan), CAPEOX (capecitabine and oxaliplatin), Bmab (bevacizumab), Cmab (cetuximab), and Pmab (panitumumab). Bold font indicates statistical significance.

**Table 2:** Surrogacy parameters of multivariate random effects models.

Model parameters	Model 1 (N = 26)	Model 2 (N = 24)	Model 3 (N = 23)
**Intercept**	−0.11 (−0.23, 0.02)	0.21 (−0.06, 0.47)	−0.04 (−0.14, 0.06)
**Slope**	**−0.22 (−0.42, −0.04)**	**0.79 (0.49–1.09)**	**0.76 (0.46–1.06)**
**Variance**	0.07 (0.04–0.13)	0.02 (0.01–0.04)	0.04 (0.02–0.07)
**R-squared**	0.24 (0.01–0.56)	0.54 (0.25–0.76)	0.53 (0.22–0.76)

Data are presented as posterior means and 95% credible intervals for the association between overall survival and overall response rate (model 1), overall survival and progression-free survival (model 2), and overall survival and both overall response rate and progression-free survival (model 3). Bold font indicates statistical significance.

**Table 3:** Comparative efficacy and safety of advanced or metastatic colorectal cancer treatments.

Heading	FOLFOX	FOLFOX+Bmab	FOLFOX+Cmab	FOLFOX+Pmab	FOLFIRI	FOLFIRI+Bmab	FOLFIRI+Cmab	FOLFIRI+Pmab	FOLFOXIRI	FOLFOXIRI+Bmab	CAPEOX	CAPEOX+Bmab
**FOLFOX**		1.84	1.16	3.07	0.63	0.46	1.00	1.36		4.94	0.45	1.71
		(0.09–8.75)	(0.29–3.19)	(0.29–12.7)	(0.22–1.44)	(0.13–1.17)	(0.27–2.64)	(0.38–3.57)		(0.19–24.7)	(0.17–1.00)	(0.08–8.37)
**FOLFOX+Bmab**	0.90		2.09	2.52	1.10	0.77	1.76	2.13		2.60	0.92	0.92
	(0.71–1.12)		(0.11–9.77)	(0.60–7.19)	(0.08–4.87)	(0.05–3.43)	(0.11–8.02)	(0.17–9.10)		(0.92–6.13)	(0.05–4.25)	(0.46–1.68)
**FOLFOX+Cmab**	1.12	1.26		3.14	0.65	0.46	0.95	1.39		5.06	0.50	1.74
	(0.92–1.35)	(0.97–1.60)		(0.31–12.9)	(0.24–1.43)	(0.15–1.07)	(0.41–1.89)	(0.43–3.43)		(0.20–25.6)	(0.14–1.29)	(0.08–8.59)
**FOLFOX+Pmab**	1.06	1.20	0.96		0.43	0.30	0.68	0.83		1.55	0.35	0.55
	(0.87–1.30)	(0.91–1.54)	(0.73–1.24)		(0.06–1.55)	(0.04–1.09)	(0.08–2.60)	(0.13–2.82)		(0.24–5.44)	(0.03–1.40)	(0.10–1.71)
**FOLFIRI**	**0.84**	0.95	**0.76**	0.80		0.73	1.61	**2.16**		7.76	0.80	2.69
	**(0.73–0.98)**	(0.76–1.18)	**(0.62–0.94)**	(0.63–1.00)		(0.38–1.26)	(0.74–3.07)	**(1.09–3.84)**		(0.39–37.4)	(0.30–1.79)	(0.16–12.5)
**FOLFIRI+Bmab**	0.95	1.07	0.85	0.90	1.13		**2.34**	**3.14**		11.3	1.21	3.93
	(0.77–1.18)	(0.88–1.29)	(0.67–1.09)	(0.69–1.17)	(0.93–1.35)		**(1.01–4.66)**	**(1.51–5.89)**		(0.56–55.2)	(0.37–3.03)	(0.23–18.4)
**FOLFIRI+Cmab**	1.06	1.20	0.95	1.01	1.26	1.13		1.50		5.46	0.56	1.89
	(0.86–1.31)	(0.92–1.53)	(0.78–1.17)	(0.76–1.32)	**(1.04–1.52)**	(0.90–1.39)		(0.56–3.32)		(0.24–27.0)	(0.17–1.41)	(0.10–9.10)
**FOLFIRI+Pmab**	0.93	1.04	0.83	0.88	1.10	0.98	0.88			3.56	0.41	1.24
	(0.74–1.17)	(0.79–1.36)	(0.63–1.09)	(0.66–1.15)	(0.90–1.33)	(0.77–1.23)	(0.67–1.13)			(0.21–16.7)	(0.12–1.05)	(0.09–5.57)
**FOLFOXIRI**	0.77	0.87	**0.69**	0.73	0.91	0.82	**0.73**	0.84				
	(0.59–1.01)	(0.63–1.17)	**(0.51–0.94)**	(0.53–1.00)	(0.72–1.15)	(0.61–1.07)	**(0.54–0.97)**	(0.62–1.12)				
**FOLFOXIRI+Bmab**	0.91	1.02	0.82	0.87	1.08	0.96	0.86	0.99	1.20		0.45	0.45
	(0.68–1.23)	(0.80–1.31)	(0.60–1.13)	(0.62–1.20)	(0.82–1.43)	(0.76–1.23)	(0.64–1.17)	(0.72–1.36)	(0.86–1.65)		(0.02–2.17)	(0.12–1.16)
**CAPEOX**	0.96	1.09	0.87	0.91	1.15	1.03	0.92	1.05	1.27	1.08		4.16
	(0.81–1.12)	(0.81–1.42)	(0.67–1.10)	(0.69–1.16)	(0.91–1.40)	(0.77–1.32)	(0.69–1.17)	(0.78–1.37)	(0.91–1.71)	(0.74–1.46)		(0.19–20.5)
**CAPEOX+Bmab**	0.95	1.06	0.86	0.90	1.13	1.01	0.90	1.04	1.25	1.05	1.00	
	(0.70–1.28)	(0.87–1.29)	(0.62–1.17)	(0.64–1.24)	(0.83–1.51)	(0.75–1.31)	(0.65–1.23)	(0.73–1.44)	(0.86–1.78)	(0.76–1.41)	(0.70–1.40)	

FOLFOX (5-fluorouracil, folinic acid, and oxaliplatin), FOLFIRI (5-fluorouracil, folinic acid, and irinotecan), FOLFOXIRI (5-fluorouracil, folinic acid, oxaliplatin, and irinotecan), CAPEOX (capecitabine and oxaliplatin), Bmab (bevacizumab), Cmab (cetuximab), and Pmab (panitumumab). Data in the left lower triangle are hazard ratios (95% credible intervals) for overall survival in the column-defining treatment with the row-defining treatment. Hazard ratios lower than 1 favor the column-defining treatment. Data in the right upper triangle are odds ratios (95% credible intervals) for adverse events grade ≥3 in the column-defining treatment with the row-defining treatment. Odds ratios greater than 1 favor the row-defining treatment. Bold font indicates statistical significance.

**Table 4:** Assumption checking of consistency and heterogeneity.

Network Meta-Analysis Assumptions	Overall Survival	Adverse Events Grade ≥3
**Consistency Assumption**		
Comparison (*p*-Value)		
FOLFIRI+Bmab vs. FOLFIRI	0.14	0.11
FOLFIRI+Cmab vs. FOLFIRI	0.08	0.29
FOLFIRI+Pmab vs. FOLFIRI	0.87	0.39
FOLFOX vs. FOLFIRI	0.93	0.81
FOLFIRI+Cmab vs. FOLFIRI+Bmab	0.24	0.35
FOLFOX+Bmab vs. FOLFIRI+Bmab	0.05	0.39
FOLFOXIRI+Bmab vs. FOLFIRI+Bmab	0.10	
FOLFOX+Cmab vs. FOLFIRI+Cmab	0.41	0.81
FOLFOX+Pmab vs. FOLFIRI+Pmab	0.63	
FOLFOX+Bmab vs. FOLFOX	0.33	
FOLFOX+Cmab vs. FOLFOX	0.42	0.82
FOLFOX+Pmab vs. FOLFOX	0.26	
FOLFOX+Cmab vs. FOLFOX+Bmab	0.96	
FOLFOX+Pmab vs. FOLFOX+Bmab	0.08	
FOLFOXIRI+Bmab vs. FOLFOX+Bmab	0.11	
FOLFOXIRI+Bmab vs. FOLFOXIRI	0.90	
**Heterogeneity Assumption**		
Global pairwise *I*^2^ (%)	28.94	71.38
Global consistent *I*^2^ (%)	36.41	76.41

FOLFOX (5-fluorouracil, folinic acid, and oxaliplatin), FOLFIRI (5-fluorouracil, folinic acid, and irinotecan), FOLFOXIRI (5-fluorouracil, folinic acid, oxaliplatin, and irinotecan), CAPEOX (capecitabine and oxaliplatin), Bmab (bevacizumab), Cmab (cetuximab), and Pmab (panitumumab).

**Table 5:** Treatment ranking probability (%) for overall survival.

Treatment	1st Line	2nd Line	3rd Line	4th Line	5th Line	6th Line	7th Line	8th Line	9th Line	10th Line	11th Line	12th Line
FOLFOX	1.29	6.79	16.79	**23.08**	**18.86**	12.94	9.11	6.17	3.50	1.19	0.24	0.04
FOLFOX+Bmab	0.14	0.78	1.86	3.32	5.24	8.11	12.12	**16.11**	**17.95**	16.65	12.31	5.40
FOLFOX+Cmab	**43.34**	**24.57**	12.78	7.57	4.80	2.87	1.77	1.09	0.65	0.37	0.14	0.06
FOLFOX+Pmab	22.17	19.43	16.95	12.07	9.75	6.67	4.70	3.36	2.41	1.40	0.77	0.33
FOLFIRI	0.00	0.01	0.03	0.14	0.49	1.89	5.37	9.98	14.96	**23.88**	**34.92**	8.33
FOLFIRI+Bmab	1.26	3.13	6.24	9.00	11.48	**15.96**	**17.42**	15.02	11.16	6.17	2.43	0.74
FOLFIRI+Cmab	16.24	**24.60**	**18.33**	12.98	10.84	6.82	4.31	2.76	1.76	0.95	0.31	0.11
FOLFIRI+Pmab	2.07	3.36	5.08	6.54	8.59	12.44	12.46	11.79	12.24	13.34	8.12	3.98
FOLFOXIRI	0.13	0.24	0.41	0.65	0.95	1.58	2.29	3.29	4.83	7.44	13.91	**64.28**
FOLFOXIRI+Bmab	3.25	3.97	5.01	5.66	6.53	8.18	9.72	11	12.18	12.35	13.93	8.21
CAPEOX	3.14	6.02	8.72	11.23	14.2	12.35	10.45	9.55	8.86	7.61	5.05	2.79
CAPEOX+Bmab	6.96	7.10	7.79	7.75	8.28	10.18	10.27	9.89	9.51	8.65	7.88	5.74

FOLFOX (5-fluorouracil, folinic acid, and oxaliplatin), FOLFIRI (5-fluorouracil, folinic acid, and irinotecan), FOLFOXIRI (5-fluorouracil, folinic acid, oxaliplatin, and irinotecan), CAPEOX (capecitabine and oxaliplatin), Bmab (bevacizumab), Cmab (cetuximab), and Pmab (panitumumab). Bold font indicates statistical significance.

**Table 6:** Treatment ranking probability (%) for adverse events grade ≥3.

Treatment	1st Line	2nd Line	3rd Line	4th Line	5th Line	6th Line	7th Line	8th Line	9th Line	10th Line	11th Line
FOLFOX	0.47	2.46	4.47	11.03	10.56	15.06	14.08	11.18	11.44	9.70	9.55
FOLFOX+Bmab	5.04	12.97	8.29	7.98	8.63	7.81	8.55	14.57	**19.83**	6.11	0.23
FOLFOX+Cmab	0.71	2.15	4.29	9.92	14.56	15.13	14.25	11.69	11.14	9.14	7.02
FOLFOX+Pmab	0.18	0.34	1.22	2.46	3.08	4.51	6.06	10.75	12.18	**31.72**	27.50
FOLFIRI	1.64	14.02	**34.98**	**18.06**	15.19	9.22	4.76	1.58	0.45	0.11	0.01
FOLFIRI+Bmab	**37.84**	**30.76**	14.14	9.38	4.81	2.04	0.68	0.25	0.08	0.02	0.00
FOLFIRI+Cmab	0.42	2.15	5.39	16.15	**18.28**	**16.84**	13.85	11.41	8.42	5.05	2.05
FOLFIRI+Pmab	0.01	0.13	0.64	3.49	7.04	13.58	**22.39**	15.35	17.52	12.73	7.11
FOLFOXIRI+Bmab	0.48	0.78	4.26	3.26	3.38	4.53	4.76	5.54	7.54	19.83	**45.64**
CAPEOX	**37.05**	23.78	14.23	9.67	6.78	3.93	2.20	1.27	0.66	0.35	0.07
CAPEOX+Bmab	16.15	10.45	8.10	8.59	7.70	7.35	8.40	**16.42**	10.74	5.27	0.82

FOLFOX (5-fluorouracil, folinic acid, and oxaliplatin), FOLFIRI (5-fluorouracil, folinic acid, and irinotecan), FOLFOXIRI (5-fluorouracil, folinic acid, oxaliplatin, and irinotecan), CAPEOX (capecitabine and oxaliplatin), Bmab (bevacizumab), Cmab (cetuximab), and Pmab (panitumumab). Bold font indicates statistical significance.

**Table 7:** SUCRA value for treatment ranking.

Treatment	Overall Survival	Adverse Events Grade ≥ 3
FOLFOX	0.65	0.40
FOLFOX+Bmab	0.33	0.50
FOLFOX+Cmab	0.88	0.42
FOLFOX+Pmab	0.77	0.19
FOLFIRI	0.19	0.71
FOLFIRI+Bmab	0.50	0.87
FOLFIRI+Cmab	0.77	0.49
FOLFIRI+Pmab	0.43	0.32
FOLFOXIRI	0.09	-
FOLFOXIRI+Bmab	0.39	0.18
CAPEOX	0.52	0.84
CAPEOX+Bmab	0.49	0.57

FOLFOX (5-fluorouracil, folinic acid, and oxaliplatin), FOLFIRI (5-fluorouracil, folinic acid, and irinotecan), FOLFOXIRI (5-fluorouracil, folinic acid, oxaliplatin, and irinotecan), CAPEOX (capecitabine and oxaliplatin), Bmab (bevacizumab), Cmab (cetuximab), and Pmab (panitumumab).
